# Pediatric dilated cardiomyopathy: a review of current clinical approaches and pathogenesis

**DOI:** 10.3389/fped.2024.1404942

**Published:** 2024-06-19

**Authors:** Ian Malinow, Daniel C. Fong, Matthew Miyamoto, Sarah Badran, Charles C. Hong

**Affiliations:** ^1^ Division of Cardiovascular Medicine, Department of Medicine, University of Maryland School of Medicine, Baltimore, MD, United States; ^2^ Department of Pediatric Cardiology, Michigan State University College of Human Medicine Helen Devos Children’s Hospital, Grand Rapids, MI, United States; ^3^ Department of Medicine, Division of Cardiology, Michigan State University College of Human Medicine, East Lansing, MI, United States

**Keywords:** pediatric dilated cardiomyopathy, infantile dilated cardiomyopathy, pediatric heart failure, cardiovascular health, echocardiography, pathogenesis, cardiomyocytes

## Abstract

Pediatric dilated cardiomyopathy (DCM) is a rare, yet life-threatening cardiovascular condition characterized by systolic dysfunction with biventricular dilatation and reduced myocardial contractility. Therapeutic options are limited with nearly 40% of children undergoing heart transplant or death within 2 years of diagnosis. Pediatric patients are currently diagnosed based on correlating the clinical picture with echocardiographic findings. Patient age, etiology of disease, and parameters of cardiac function significantly impact prognosis. Treatments for pediatric DCM aim to ameliorate symptoms, reduce progression of disease, and prevent life-threatening arrhythmias. Many therapeutic agents with known efficacy in adults lack the same evidence in children. Unlike adult DCM, the pathogenesis of pediatric DCM is not well understood as approximately two thirds of cases are classified as idiopathic disease. Children experience unique gene expression changes and molecular pathway activation in response to DCM. Studies have pointed to a significant genetic component in pediatric DCM, with variants in genes related to sarcomere and cytoskeleton structure implicated. In this regard, pediatric DCM can be considered pediatric manifestations of inherited cardiomyopathy syndromes. Yet exciting recent studies in infantile DCM suggest that this subset has a distinct etiology involving defective postnatal cardiac maturation, such as the failure of programmed centrosome breakdown in cardiomyocytes. Improved knowledge of pathogenesis is central to developing child-specific treatment approaches. This review aims to discuss the established biological pathogenesis of pediatric DCM, current clinical guidelines, and promising therapeutic avenues, highlighting differences from adult disease. The overarching goal is to unravel the complexities surrounding this condition to facilitate the advancement of novel therapeutic interventions and improve prognosis and overall quality of life for pediatric patients affected by DCM.

## 1 Introduction

Pediatric Dilated Cardiomyopathy (DCM) is a cardiac condition characterized by systolic dysfunction with bi-ventricular and atrial dilatation with reduced myocardial contractility (1). Most pediatric DCM patients initially present with symptoms of congestive heart failure with associated electrical conduction disturbances or extra-cardiac abnormalities. Adults develop DCM as well, secondary to lifestyle, genetics, and environmental exposures. There exists a robust body of evidence supporting mortality-improving treatments in adult DCM. The same basis of evidence does not exist for pediatric DCM and much of treatment is extrapolated from studies on adults. This paper focuses on the clinical and molecular distinctions of the pediatric variant of DCM offering guidance into diagnostic approaches, treatments plans, and research discoveries within the field.

### 1.1 Purpose of review

This paper aims to offer a comprehensive review of pediatric DCM, focusing on both clinical and biological aspects of the disease. The significance of this review is underscored by the current limited treatment options and poor prognosis for patients diagnosed with pediatric DCM. This review highlights recent findings within the field to shed light on the complex pathophysiology of pediatric DCM and its genetic underpinnings. The collective goal is to demystify this largely idiopathic disease, offering hope for novel treatments and interventions to improve the outcomes and quality of life for affected children.

## 2 Epidemiology

The prevalence of pediatric DCM is reported to be 0.57–1.13 cases per 100,000 individuals and account for approximately 50% of all pediatric cardiomyopathies (2, 3). In infants less than one year old, the prevalence rises to a striking 8.34 cases per 100,000. This condition exhibits variations in prevalence across demographics (per 100,000), with males (1.32) more affected than females (0.92) and black patients (1.47) showing a higher rate of disease than the white patients (1.06) (3). Geographical disparities were also noted in a study with New England region (1.44) having a higher prevalence than the Central Southwest region (0.98) (4). These figures may represent an underestimation of actual disease prevalence due to unexplained sudden deaths, emphasizing the importance of further research into this condition (3).

Pediatric DCM predominantly affects children in the first two years of life, with a median age of diagnosis at 1.5 years, and 41% of patients being diagnosed within the first year (3, 4). The prognosis for children diagnosed with pediatric DCM is grim, with nearly 40% undergoing heart transplantation or dying within two years of diagnosis (3). Additionally, the estimated financial burden to care for pediatric DCM patients in the US is $4–10 billion spent annually (5, 6).

Pediatric DCM may develop as a primary congenital condition or derive secondary another malady that stresses the heart. Common etiologies include inborn errors of metabolism, syndromic causes, myocarditis, and familial cardiomyopathy (4). Inflammation, toxicities, or inherited pathogenic variants may also be central to DCM development (7). The majority of pediatric DCM cases (66%–69%), however, remain idiopathic (8, 9). These idiopathic cases are largely believed to stem from underlying genetic abnormalities (10).

The epidemiologic data in pediatric DCM differs from that seen in adult DCM. For example, the incidence of DCM is 10-fold higher in adults (3, 4, 11). Critical differences in the development of DCM account for these distinctions. First, children have less exposure to chronic health habit-associated risk factors that commonly lead to adult DCM. Co-morbidities, which are less common in children, often impose damage to cardiomyocytes in adults. Second, there is a longer latency period for the clinical expression of the effects of genetic and environmental factors on the heart. These factors are more evident in adults due to their wider age span compared to pediatrics (≤18 years old). This extended duration of exposure provides adults with more opportunities to develop DCM (3).

## 3 Clinical presentation

Understanding the multifaceted clinical features of pediatric DCM is essential for healthcare providers to expedite diagnosis, institute timely treatment, and provide appropriate support for pediatric DCM patients. Children with DCM manifest along a spectrum of disease severity. A review of key findings from the first 15 years of the Pediatric Cardiomyopathy Registry and Heart Failure (PCMR): noted 71% of patients with Pediatric DCM present with signs and symptoms of congestive heart failure (CHF) and 27% are further classified as NYHA Class IV heart failure (12). Symptoms can include dyspnea, feeding difficulties and, in the most severe cases, sudden cardiac death (SCD). In the National Australian Childhood Cardiomyopathy Study (NACCS), which followed a cohort of 184 children with pediatric DCM from 1987 to 1996, it was found that 5% of cases are diagnosed following sudden cardiac death. Another 2% were identified due to exercise intolerance or arrhythmias and 3% were detected by routine screening in those with family history. On presentation to the hospital, 45% of the NACCS patients required intensive care unit (ICU) admission. Given the compromised cardiac function, clinicians deemed inotropic support necessary in approximately 40% of cases to maintain adequate circulation and perfusion. Additionally, clinical courses of some patients were complicated with disease-related comorbidities including renal failure, thromboembolic events, and hepatic impairment (13). These data highlight the need for vigilance in evaluating seemingly benign symptoms in children to promote early detection and prevent acute CHF exacerbations or SCD.

## 4 Pathologic & histologic findings

The pathological characteristics and histological findings of pediatric DCM unveil a complex landscape of structural alterations within the myocardium. Grossly, the chambers of the heart are dilated, giving it a globular shape and increased weight. The ventricular trabeculations of affected patients are notably stretched and thin. The left ventricular (LV) endocardium and myocardium undergo significant fibrotic thickening. Patchy fibrosis is a common finding and may derive from inflammatory etiologies, such as myocarditis. Taken together, these structural changes lead to impaired contractility and diminished compliance. Additional clinical risk can be attributed to the fibrotic changes creating a propensity for mural thrombus formation and compounding the risk of thromboembolic events (1).

Histological characteristics seen in pediatric DCM include nuclear enlargement, hyperchromasia, and intimal fibroelastic thickening. Individual myocytes are stretched, thin, and wavy. Inflammatory cell infiltrates may be seen on histology depending on the etiologic basis of a patient's DCM. Inflammatory cells, particularly lymphocytes, present in significant quantities within a sample, along with appropriate clinical context, suggest possible myocarditis and should prompt further investigation for the presence of viruses. Other inflammatory changes could also drive these histologic findings and must be considered. Fibrotic changes that result from these inflammatory processes can subsequently alter cardiac function (1). The intricate pathological and histological features of pediatric DCM offer clues into the mechanistic processes & adaptive strategies driven by disease.

## 5 Diagnostic methods

Current diagnostic methods and tools employed in clinical practice aim to supplement a thorough clinical history & physical exam and provide valuable insights into the structural and functional abnormalities of the heart in affected pediatric patients.

The 1995 World Health Organization/International Society and Federation of Cardiology Task Force defined dilated cardiomyopathy as dilatation and impaired contraction of the left ventricle or both ventricles. It clarifies that the degree of myocardial dysfunction must be independent of abnormal loading conditions or extent of ischemic damage (14). The American Heart Association (AHA) published a definition in 2019 specific to pediatric DCM, which maintains the same essence, stating that pediatric DCM consists of a dilated LV with systolic dysfunction. It further stipulates that the dilation and dysfunction seen should not be secondary to physiologic or anatomic hemodynamic changes associated with abnormal loading conditions or ischemia, unless the morphofunctional DCM phenotype is retained after appropriate intervention (2). These defintions aim to emphasize the intrinsic impairment of cardiomyocyte contractile function in the LV and exclude many secondary processes that also cause LV dilation and systolic dysfunction. The phenotype specific to LV dilatation with associated systolic dysfunction in the absence of other reversible or treatable pathology is critical in influencing clinical decision-making. Dilatation specifically refers to left ventricular end-diastolic diameter (LVEDD), while the systolic dysfunction is in reference to EF or fractional shortening (15, 16).

Echocardiography is the fundamental diagnostic tool for the initial evaluation of pediatric patients presenting with symptoms suspicious of DCM. The diagnostic guidelines provide absolute cutoffs for a disease that varies widely in severity. The diagnostic criteria enumerated in a statement from the AHA detail LVEDD z-score >2 and left ventricular end-systolic diameter (LVESD) z-score >2 as determined by echocardiography. The patient's EF can subsequently help determine the degree of systolic dysfunction (2). While the guidelines provide an adequate basis for diagnosis, other echocardiogram parameters influence a clinician's understanding of cardiac function. Notably, in a study involving 1,426 pediatric patients diagnosed from 1990 to 2003, echocardiography demonstrated a diminished fractional shortening with z-score (−9.16), an increased LVEDD Z-score (4.17), an elevated LV mass Z-score (2.34). DCM patients LV end-diastolic posterior wall thickness and septal wall thickness were found to be comparable to those of non-DCM patients (3). The clinically important echocardiographic finding is impaired LV function as evidenced by diminished fractional shortening, ejection fraction (EF), LVEDD size, and hypokinesis, among other markers to indicate the presence and degree of dysfunction.

Cardiac Magnetic Resonance (CMR) is another valuable diagnostic tool, although diagnosis of pediatric DCM generally does not require its usage. CMR offers a comprehensive assessment of cardiac structure and function and is particularly useful for determining the presence and pattern of fibrosis. In most situations, patients present with symptoms of pediatric DCM. These symptoms are sequelae of cardiac dysfunction and, therefore, echocardiography should be sufficient to establish a diagnosis. There are certain instances, however, where CMR reveals developing cardiac dysfunction in advance of symptoms or echocardiographic changes. Neuromuscular disorders are one such example. A cohort of Becker muscular dystrophy (BMD) underwent cardiac evaluation by echocardiogram & CMR with late gadolinium enhancement (LGE), a technique that shows regions of myocardial fibrosis. With echocardiogram imaging, only 53% showed impaired LVEF, while with CMR imaging, 73% showed evidence of LGE (17). Duchenne muscular dystrophy (DMD) patients also showed LGE on CMR before echocardiogram changes were found (18, 19). By technical definition, without changes to cardiac physiology, these would not yet be classified as DCM. However, given the imminent development of pediatric DCM with these progressive diseases, measures to prevent or delay adverse cardiac remodeling must be considered. Strain analysis is another technique that has shown promise on echocardiogram and CMR in detecting maladaptive physiology before symptoms present (20). Additionally, while CMR is not often necessary in the initial diagnosis of pediatric DCM, it can provide insight into the etiology, including myocarditis, inflammation, myocardial edema, and iron overload (21). Other modalities for diagnosis are not often needed and should be selected on a patient-specific basis.

The definition and diagnostic criteria of pediatric DCM provide absolute measures which are useful for classification of patients as diseased or not. Absolute measures for pediatric DCM, however, oversimplify a complex disease and should not substitute for the nuance observed in clinical practice. These metrics are meant to provide basic framework from which clinicians must consider additional factors to assess whether a child is at risk for symptoms, progression, or complications related to cardiac dysfunction.

## 6 Screening

Screening for pediatric DCM facilitates early detection and intervention to avert adverse outcomes. Echocardiography is the most utilized screening method recommended in individuals with a familial history of DCM, inborn errors of metabolism, neuromuscular disorders, and malformation syndromes associated with DCM (7). The Heart Failure Society of America in collaboration with the American College of Medical Genetics and Genomics offers screening recommendations that are tailored to different age groups and risk profiles. For children with a first-degree relative affected by DCM, the guidelines suggest annual screening for children aged 0–5 years, followed by screening every 1–2 years for those aged 6–12, and every 1–3 years for children aged 13–19 (22). A study examining the family members of 59 patients with idiopathic pediatric DCM demonstrated that 20.3% of families had at least one family member who displayed echocardiographic findings consistent with DCM (23). Given these results, the Heart Rhythm Society (HRS) and the European Heart Rhythm Association (EHRA) recommend genetic testing of at-risk first-degree relatives. Therefore, genetic testing and echocardiogram screening of at-risk first-degree family members of index cases is recommended. Individuals with idiopathic disease should undergo genetic testing for potential causative mutations as well (24). The list of genetic mutations associated with pediatric DCM continues to expand rapidly and the genetic panels should include mutations reflecting this growth.

## 7 Cellular distinctions between pediatric & adult dilated cardiomyopathy

Pediatric dilated cardiomyopathy and its adult counterpart exhibit distinctions at the clinical and molecular levels. DCM is much more prevalent in adults, which reflects the cumulative effects of co-morbid disease and lifestyle & environmental exposures. From a diagnostic perspective, the approach to each patient requires vigilance for common heart failure presentations matched with relevant echocardiographic data. Treatment options employed by physicians are similar among children and adults; however, their efficacies in each group are different. These differences are accounted for by the distinct molecular pathways and histopathological changes as a child's hearts adapt to the dysfunctional physiology imposed by DCM. By emphasizing these distinctions, the aim is to demonstrate the basis of how clinical management of pediatric DCM patients should differ from that of adults.

### 7.1 Molecular distinctions

A study examining explanted hearts from children and adults with idiopathic DCM demonstrates a downregulation of β_1_ and β_2_ adrenergic receptors on cardiomyocytes, whereas adults maintain β_2_ receptor expression. This β_2_ pathway is known to have both cardiostimulatory & cardioinhibitory actions that affect cardiac remodeling (25, 26). Along the β-adrenergic pathway there is also a decrease in cyclic adenosine monophosphate (cAMP) levels in both pediatric & adult DCM, however, the extent of decrease varied. The cAMP levels in children remained significantly higher than those in adults. Additionally, there is an upregulation of connexin43 expression in pediatric patients and a corresponding downregulation in adults (27). Connexin43 encodes a gap junction protein critical for the passage of small ions & metabolites between neighboring cardiac cells (28). No change in phosphatase expression is seen in pediatric DCM, while there is an increase in adults (27). The specific phosphatases studied, PP2A & PP1β, play a role in the excitation-contraction coupling of cardiac muscle (29). Furthermore, decreased phosphorylation at the Ser16 or Thr17 sites of phospholamban was not found in pediatric hearts. Phospholamban is a key regulator of sarcoendoplasmic reticulum calcium ATPase (SERCA) activity & cardiac contractility, known characteristics that disrupt healthy physiology in adult heart failure (27, 30).

At the gene expression level, mRNA profiles also differ with specific genes showing age-dependent expression patterns. RNA sequencing of DCM cardiomyocytes showed a 2-fold increase in 387 mRNA in children compared to 223 mRNA in adults. Genes known to be involved in pathologic cardiomyocyte hypertrophy, such as *NPPA*, *MYH7*, *MYBPC3*, *ACTC1*, *TPM1*, *DES*, *LMNA*, *MYL3*, *MYLK3*, *PLN*, *GDF15*, and *MEF2*, were found to be upregulated in adults. Further, *MYH6*, another contributory gene that decreases pathologic cardiomyocyte hypertrophy was downregulated in adults as compared to children with DCM. This study then proceeded to investigate molecular pathways that are known effectors of cardiac remodeling in DCM. In adult DCM, there was upregulation of oxidative reduction, fatty acid metabolism, responses to endogenous stimuli & organic substances, and inflammatory wound response pathways. In pediatric DCM, pathways involved in cell adhesion, ion transport, transmembrane transport, and visual perception were upregulated. These differences are directly reflective of more robust adaptive capabilities of young cardiac tissue.

### 7.2 Histopathological distinctions

Pathological changes in pediatric DCM are distinct from those in adult DCM. Children have been shown to experience less adverse cardiac remodeling compared to adults as measured by cardiac fibrosis, cardiomyocyte hypertrophy, inflammation, and capillary loss (31–33). Children with DCM exhibit no significant change in myocyte cross-sectional area and sarcomere thickness, while adults demonstrate an increase in each (33). Histological examinations reveal distinct patterns of fibrosis. Pediatric patients predominantly exhibit patchy fibrosis within the myocardium compared to an accentuated perivascular fibrosis in adult populations. Further, pediatric patients exhibit a significantly higher myocarditic index, a value influenced by the severity of interstitial fibrosis, size variation of myocytes, disarrangement of muscle bundles, and amount of mononuclear cell infiltration (34). Separate inflammatory processes and structural derangements within the myocardium may explain this difference. Pediatric patients with poor prognosis were found to have more myocytolysis and/or fragmentation of muscle bundles. Further, myocardial hypertrophy with disorganization was seen in 30% of pediatric patients and none of the older adult patients. These distinctions suggest disease-related variations in DCM pathogenesis.

Understanding these pathologic processes that occur pediatric DCM and adult DCM is paramount for tailored research endeavors, clinical management, and therapeutic strategies. The evidence presented above stresses the need for further exploration into the molecular physiology at play in the response of pediatric cardiac tissue to DCM. Deeper insight into the complex interplay of molecular, clinical, and pathological differences will then direct more effective age-specific therapeutic approaches to DCM.

## 8 Etiology

The pathogenesis of pediatric dilated cardiomyopathy encompasses a diverse array of causative and etiological factors. These range from genetic predispositions causing primary disease to inflammatory & metabolic derangements leading to secondary DCM. Any condition that contributes to significant LV volume stress in children can account as a causative factor given the nature of disease pathogenesis, so this review will focus on the more notable etiologies (Table 1). In a comprehensive examination of 261 cases, the etiological distribution was as follows: Idiopathic cases accounted for the majority at 76%, indicating that the precise cause remains unknown in a significant proportion of cases. Myocarditis was the cause in 12% of cases, highlighting the role of inflammatory processes in the development of DCM. Familial isolated cardiomyopathy was responsible for 7% of cases, emphasizing the hereditary component in disease development. Neuromuscular disorders contributed to 3% of cases, with a notable presence of conditions such as Duchenne and Becker muscular dystrophy. Metabolic factors were implicated in 1% of cases, emphasizing the significance of inborn errors of metabolism in a small subset of patients. Additionally, DCM was found to co-occur with recognized malformation syndromes in 1% of cases, underscoring the potential interplay of genetic factors. Among this group, a family history of cardiomyopathy was present in 26% of cases while 13% had a family history of sudden cardiac death, indicating the necessity for early detection to drive early preventative interventions (35).

**Table 1:** Major etiologies of pediatric dilated cardiomyopathy. Singh et al. (35), Towbin et al. (3).

Etiology	Singh et al. (*n* = 261)	Towbin et al. (*n* = 1,426)
Idiopathic	76%	66%
Myocarditis	12%	16%
Familial cardiomyopathy	7%	5%
Neuromuscular disorders	3%	9%
Inborn errors of metabolism	1%	4%
Syndromic	1%	1%

In a larger cohort of 1,426 cases, the etiological distribution revealed similar trends. Idiopathic cases accounted for the majority, representing 66% of the cases. Myocarditis was responsible for 16% of cases and neuromuscular diseases, primarily Duchenne and Becker muscular dystrophy, contributed to 9% of cases. Familial DCM was the cause in 5% of cases, and the genetic basis was further delineated, with 68% following an autosomal dominant inheritance pattern. Further genetic testing revealed two families with delta-sarcoglycan gene mutations and two families with Z-band alternatively spliced PDZ domain protein (ZASP) gene abnormalities. Further characterization of familial pediatric DCM displayed 24% of disease is related to autosomal recessive inheritance and 2% is X-linked inheritance. Tracking causative mutations of X-linked inheritance showed one patient with a dystrophin mutation and another with a tafazzin mutation. Additional gene mapping suggested 6% of familial DCM patients display a complex phenotype of LV noncompaction (3). Regarding inheritance, mitochondrial inheritance can also play a significant role in the development of pediatric dilated cardiomyopathy (1). Inborn errors of metabolism were responsible for 4% of cases with 46% of these patients displaying mitochondrial disorders, 24% with Barth syndrome, and 11% displaying systemic carnitine deficiencies. Additionally, 1% of cases were associated with malformation syndromes, with Alström syndrome (33% of syndromic patients) being a prominent contributor (3). Cardiotoxic agents, particularly anthracyclines, are another source that can potentiate DCM disease in children. Other etiologies that present with LV dilatation and systolic dysfunction are critical to consider in the appropriate clinical contexts including endocrine diseases, autoimmune diseases, infiltrative diseases, and nutritional deficiencies (36, 37). These findings illustrate the wide range of etiologic influence on DCM. Prognosis of disease is impacted by these factors and therapy should be assigned accordingly. With further insight into the genetic pathogenesis of pediatric DCM, targeted therapies can contribute to improved prognosis.

## 9 Genetics

The genetic landscape of pediatric dilated cardiomyopathy encompasses a broad spectrum of mutations that play a pivotal role in the onset and progression of this disease. These genetic underpinnings outline the initial malady of a cascade toward phenotypic presentation of pediatric DCM. The most frequent mutations impact the sarcomere, cytoskeleton, sarcolemma, ion channels, or intercellular junctions (10). Prevalence and time of phenotypic appearance of each gene varies. Many of these proteins account for manifestations of pediatric or adult DCM depending on specific mutations and extent of phenotypic expression. The following discusses a select list of mutations that have been discovered to contribute to pediatric DCM (Table 2), but many others exist and even more have yet to be discovered. Additionally, the subdivision of proteins by purpose is fluid as many proteins help multiple systems function properly.

**Table 2:** Select proteins implicated in disease pathogenesis of pediatric dilated cardiomyopathy.

Protein family	Implicated genes
Sarcomeric proteins	Actin: *ACTC1*, *ACTC2*
	Actin-binding protein: *ACTN2*, *FLNC, NEXN*
	Myosin: *MYH6, MYH7*
	Myosin-binding protein: *MYBPC3*
	Titin: *TTN*
	Tropomyosin: *TPM1*
	Troponin: *TNNT2*, *TNNC1, TNNI3*
Nuclear protein	Lamin: *LMNA*
	Thymopoietin: *TMPO/LAP2*
Ion channels & transport proteins	Junctophilin: *JPH2*
	Phospholamban: *PLN*
	Sodium Channel: *SCN5A*
Cytoskeletal & ECM proteins	Desmin: *DES*
	Desmoplakin: *DSP*
	Dystrophin: *DMD*
	Fukutin: *FKT*
	Laminin: *LAMA4*
	LIM domain binding protein: *LDB3*
	Metavinculin: *VCL*
	Rotatin: *RTTN*
Other Proteins	BCL2-associated protein: *BAG3*
	RNA-binding motif protein: *RBM20*

### 9.1 Sarcomeric proteins

Sarcomere gene mutations are estimated to contribute to 35%–40% of pediatric DCM cases (38). In a 2011 study reviewing evidence of pediatric DCM genes, several have been identified with compelling evidence of their involvement. These genes include *TPM1* (encoding tropomyosin), which is associated with 32% of cases, and *TNNT2* (encoding cardiac troponin T), implicated in 21% of cases. Additional genes encoding cardiac sarcomere or cytoskeletal proteins, such as *MYBPC3*, *MYH6*, *MYH7*, *TNNC1*, and *TNNI3*, were seen to be significantly associated with DCM (39). There are many prominent sarcomeric proteins implicated in familial disease—with varying levels of evidence—acting on different parts of the sarcomere. These include myosin proteins (MYH6 (40), MYH7, MYBPC3 (41)), actin proteins [ACTC1, ACTC2 (42)], tropomyosin protein [TPM1 (43)], cardiac troponins (TNNT2 (44), TNNC1 (45), TNNI3 (46)), actin-binding proteins (actinin a2 (47), filamin C (48), nexilin F-actin-binding protein (49, 50)). Sarcomeric protein mutations understandably limit cardiac force generation and transmission, contributing to eventual volume overload and subsequent DCM (51, 52). The *TTN* gene, for example, is a sarcomeric gene responsible for encoding titin. A study examining its role in DCM development reported truncation was found in 25% of familial DCM cases and 18% of idiopathic DCM within their study population. Titin presence in the sarcomere is critical to maintenance of passive force and elasticity necessary for proper diastolic and systolic function. Inadequate function leads to pediatric DCM sequelae (53).

### 9.2 Nuclear proteins

*LMNA* and *TMPO* (*LAP2*), are implicated in −8% and −1% of cases, respectively (54, 55). These genes encode nuclear envelope structures. The pathogenesis of nuclear protein mutations to development of DCM is not well understood. Lamin A and C are known to have diverse cellular functions. One proposed mechanism suggests that *LMNA* mutations cause cellular and tissue fragility, especially in mechanically stressed tissues like the heart and muscles (56). A-lamins also play essential roles in chromatin organization, signal transduction, differentiation, repair, and anchoring lamin-binding proteins (57). Disruptions in any of these processes can lead to cell damage, apoptosis, and impact cardiac and muscle function.

### 9.3 Ion channels & transport proteins

Genes like *SCN5A* and *PLN* are associated with sodium channel function and calcium pump inhibition (58, 59). *SCN5A* is noted to contribute to 1.7% of cases of familial DCM among the Familial Cardiomyopathy Registry (60). Junctophilin-2 mutations (*JPH2*) disrupt interactions between the cardiomyocyte sarcolemma and sarcoplasmic reticulum, necessary for efficient calcium release in excitation-contraction coupling. Improper ion concentration intracellularly or improper transfer of ions between cells will lead to weak or dyssynchronous contractions. Impaired contractility leads to muscular remodeling of the LV that progresses to DCM.

### 9.4 Cytoskeletal & extracellular matrix proteins

Proteins involved in structural integrity of individual cardiomyocytes and cardiac tissues can be contributory to disease development. Mutations in the desmin protein (*DES*) disrupt the structural integrity of cardiac muscle cells, leading to myofibrillar disarray and cell damage (61). *LDB3* mutations (Cypher/ZASP proteins) impact sarcomere assembly, leading to impaired contractility and myofibrillar disorganization (62). Dystrophin mutations (*DMD*) weakens the structural stability of cardiac muscle cells, making the heart more susceptible to DCM (63). Metavinculin mutations (*VCL*) disrupt the linkage between the sarcomere and the sarcolemma, leading to impaired contractile function and cellular damage (64). Disruptions of information sensing extracellular matrix proteins can affect signal pathways as seen with *LAMA4* (Lamin α4) and *FKT* (Fukutin) mutations (65, 66). Mutations in the desmoplakin gene (DSP) disrupt desmosomes, leading to weakened cell-cell adhesion and structural instability in cardiac muscle cells (67). Recent discoveries linking dysfunctional centrosomal reduction to pediatric DCM has led to consideration of a novel set of proteins involved in the disease. During maturation cardiomyocytes shift their microtubule-organizing complexes from the centrosome to its perinuclear location. Gene mutations encoding proteins that direct this reorganization, such as the studied *RTTN* mutation, leads to misaligned microtubules, and thus impaired cardiac contractility (68). The exact pathogenesis of these mutations is incompletely understood, but from a large-scale perspective, impaired structure will prevent optimal force generation. This process leads to ventricular fluid retention developing into eventual DCM.

### 9.5 Other proteins

BAG3 (BCL2-associated athanogene 3) is an anti-apoptotic protein located at the Z-disc of cardiac muscle cells. It plays a critical role in preventing apoptotic cell death induced by mechanical stress, a vital function in maintaining cardiac structural integrity (69, 70). RBM20 (RNA-binding motif protein 20) is involved in RNA splicing and has been associated with DCM development. Mutations in RBM20 can lead to aberrant splicing of cardiac genes, affecting the function of key cardiac proteins and contributing to DCM pathogenesis in both pediatric and adult populations (71).

The mechanisms by which individual genetic mutations disrupt normal physiological function exhibit substantial variability. Nevertheless, these distinct pathways collectively contribute to the pathogenesis of pediatric dilated cardiomyopathy (DCM). A theoretical construct proposed by Towbin and Bowles postulates the existence of a “final common pathway” that serves as a pivotal point in the pathogenesis of DCM associated with various genetic mutations. In essence, this concept suggests that the commonality among these diverse mutations lies in the impairment of the transmission of contractile forces from the sarcomere through the extracellular matrix, consequently compromising the normal physiologic response of sarcomeres to stretch (72). The elucidation of underlying pathogenic mechanisms remains a dynamic field of study. Research into these pathways open new avenues for understanding the pathogenesis of idiopathic dilated cardiomyopathy. Emerging perspectives offer fresh insights into the intricate web of genetic factors underpinning the condition and, in time, may offer novel targets for therapeutic approaches. These findings underscore the importance of continually advancing our understanding of the genetic determinants of pediatric DCM.

## 10 Infantile dilated cardiomyopathy

Infantile dilated cardiomyopathy (iDCM), a subset of pediatric dilated cardiomyopathy, is characterized by presentation of heart failure during the perinatal period. Presentation of DCM during this window has been associated with poor prognoses and many of these patients will eventually require a heart transplant (2, 38, 73–75). Thus, there is a significant need to understand the etiology of iDCM to aid in diagnosis, guide genetic counseling, and develop therapeutics.

A well-studied biological process that is co-occurring during this perinatal window, known as cardiomyocyte maturation, is characterized by a sequence of structural and functional changes which equip the cardiomyocyte to support postnatal life (i.e., cardiomyocyte hypertrophy, sarcomere organization, cessation of proliferation) (76). Significant interest has been invested into understanding this process in the context of stem cell derived cardiomyocytes, as impaired maturation limits their utility in cell therapy and disease modeling (77–79); however, whether defects in this process are linked to human cardiac disease is relatively under studied.

Based on the temporal correlation between iDCM presentation and onset of cardiomyocyte maturation, our group hypothesized that maturation may be implicated in at least a subset of cases of iDCM. The Hong lab recently identified a patient who presented as an infant with heart failure and was eventually diagnosed with an isolated, non-syndromic case of iDCM (68). Through use of various sequencing approaches, stem cell derived cardiomyocyte and *in vivo* models, causal compound heterozygous mutations were identified in a gene called *RTTN*, which encodes the protein Rotatin, a component of the centrosome. Interestingly, maturation of cardiomyocytes with these mutations were notably immature, likely secondary to the affected centrosomes and cytoskeletal disarray. This data leads to consideration that immature cardiomyocytes were underlying the patients iDCM, offering a potential therapeutic avenue if such cardiomyocytes would be able to be coaxed into maturity. Previous work looking into Alström syndrome provides support to this hypothesis. A study which investigated mutation of the centrosome gene *ALMS* showed increased cell cycling of cardiomyocytes, perhaps also suggestive of impaired maturation in Alström syndrome related iDCM (80). Continuing efforts into understanding the cardiomyocyte maturation process, in particular how the centrosome is involved, may aid in elucidation of the key pathogenic mechanisms behind iDCM, pediatric, and adult DCM.

## 11 Prognosis

A complex interplay of clinical, genetic, and therapeutic factors contributes to varying prognoses of pediatric DCM. These distinct aspects of individual DCM disease lend insight into the more personalized therapeutic strategies. Broadly, the degree of ventricular dysfunction and cardiac remodeling is the central factor through which other variables affect prognosis.

Aspects of disease predictive of disease normalization, survival, transplant, and death were researched across a spectrum of pediatric DCM patients. A study of 741 DCM individuals in the PCMR demonstrated that younger children and those with lower LVEDD z-scores were found more likely to normalize to non-DCM cardiac function at 2 years after diagnosis (81). A systematic review of clinical studies following outcomes of pediatric DCM patients noted consistent findings of greater survival in those with younger age a diagnosis, higher LV fractional shortening, higher LVEF, elevated left ventricular end-diastolic pressure (LVEDP), and presence of myocarditis (82). Increased LVEDD was a significant predictor of increased cardiac transplantation but not death (83). Conversely, short stature was predictor of death but not transplantation (9). Clinical predictors of death from idiopathic DCM included dyspnea, reduced pedal pulses, and NYHA functional class IV. Predictors of death based on imaging technique included maximal cardiothoracic ratio and pulmonary congestion on chest x-ray; right atrial overload, ventricular arrhythmias, and elevated heart rate (HR) on ECG; and grade 3 or 4 mitral regurgitation, increased left atrium/aortic ratio, and lower LVEF on echocardiography (83).

The only definitive treatment of a failing DCM heart is transplantation. Therefore, disease prognosis can be highly impacted by time of listing and organ availability. A study merged the PCMR and the Pediatric Heart Transplant Study (PHTS) pediatric DCM databases to investigate these factors. Of 261 children included, the median age at diagnosis is 2.5 years old with 43.1% diagnosed within the first year of life. The median age at the time of listing is 3.7 years old and the median age at the time of transplant is 5.4 years old. After diagnosis, listing took place within a median of 1.9 months. Of those listed, 80% of patients underwent transplant by a median of 0.8 months. At the time these children with DCM were listed for transplant, 92% were hospitalized, 71% were on IV inotropes, 30% required assisted ventilation, 9% were on extracorporeal membrane oxygenation (ECMO), and 4% were using a ventricular assist device (35). While waiting for transplant, 11% (29/261) patients died, predominantly from multiorgan failure and CHF (84). Inherently, there are risks to transplantation as well with 13 patients dying within 6 months of their transplant operation (35).

A separate cohort including 1426 DCM patients, determined similar median ages of diagnosis, listing, transplant, and death (1.5 years old, 4.0 years old, 4.8 years old, and 3.0 years old, respectively). These data can be contextualized by considering the occurrence of heart transplantation at 1, 3, and 5 years after diagnosis, which stands at 22%, 27%, and 29%, respectively (85). Among 1,199 pediatric DCM patients in the 1990s and 754 patients in the 2000s recorded in the PCMR database, the rates of normalization, as determined by echocardiography, were 30% and 27%, respectively, while the rates of transplantation were 24% for both decades. Similar results were seen in children with the idiopathic subtype of DCM with 22% recovering normal LV function, 27% having persistent disease, and 51% undergoing transplantation or death (81).

At 12 months after listing for transplant the mortality among patient differed by prior therapy. A 26% mortality was found for those on mechanical ventilation or circulatory support compared to 16% on inotropes and a 9% mortality when neither therapy was required (86). Prior therapy, however, is likely a marker of disease severity. Nonetheless, disease severity has a notable impact on survival. Undergoing transplant, however, dramatically alters prognosis. After transplant the survival rate at 1-year is 92% and at 5-years is 80% (86, 87). Transplant, of course, is not without inherent danger and limitations.

The overall survival rates for pediatric DCM at 1, 2, 5, and 10 years from diagnosis were determined to be 87%, 83%, 77%, and 70%, respectively (3). These survival rates represent those of pediatric DCM patients independent of etiology and interventions. Different etiologies, however, impart varying prognosis related to pathologic course, disease comorbidities, and therapeutic options. A review of prevalent causes of pediatric DCM showed 5-year survival of familial DCM (94%), myocarditis (92%), inborn errors of metabolism (86%), and neuromuscular disorders (57%) (3). Similar distinctions exist for transplant-free survival data (Table 3) (88).

**Table 3:** Etiology-based transplant-free survival rates of pediatric DCM patients at 1-year, 5-year, 10-year, respectively (88).

Pediatric DCM etiology	*n*	1-year survival	5-year survival	10-year survival
All	1,426	69%	54%	46%
Idiopathic	941	61%	47%	42%
Myocarditis	222	79%	73%	60%
Neuromuscular disorder	125	83%	52%	26%
Familial	66	81%	59%	59%
Inborn error of metabolism	54	84%	78%	78%
Marfan syndrome	15	91%	76%	76%

Sudden cardiac death (SCD) is a particularly feared complication of pediatric DCM. A study of 1,803 pediatric DCM patients, the 5-year rate of non-SCD-related deaths was 12.1%, while a notable 2.4% experienced SCD. Further evaluation of the SCD group demonstrated higher rates of CHF at presentation, increased anti-arrhythmic therapy use at 1 month, and smaller LV posterior wall thickness (85). These distinctions prompt potential specialized therapeutic consideration of AICDs in patients at risk. A review of the PCMR data displayed death from SCD in 12% of patients. While this percentage of SCD occurring merits strong preventative efforts, this value is significantly less than that of adults with non-ischemic DCM (12).

The prognosis of pediatric DCM is influenced by the age of onset, the degree of structural disease at presentation, and disease etiology. Prognostic factors encompass a wide range of clinical and diagnostic indicators. Advancements in resuscitation, supportive therapies, and/or adult-based chronic heart failure treatments are believed to have contributed to improving survival rates in pediatric DCM over time (89). A comprehensive understanding of these prognostic factors is essential for accurate communication with families and determining optimal management of pediatric DCM patients.

## 12 Treatment strategies

The objective in treating pediatric dilated cardiomyopathy is threefold: (1) manage congestive heart failure symptoms, (2) mitigate progression of structural heart disease, and (3) avert life-threatening arrhythmias. The AHA guidelines for treating pediatric heart failure with reduced ejection fraction (HFrEF) provide a comprehensive framework to assist with therapeutic decision-making. These guidelines categorize treatment modalities based on the degree of evidence specific to children (pediatric DCM relevant recommendations are in Supplementary Table S1) (90). Certain individuals may necessitate deviation from therapeutic guidelines based on patient-specific factors. It is imperative to emphasize the scarcity of randomized controlled trials in the realm of pediatric DCM treatment, primarily attributable to low disease frequency.

Irrespective of guidelines, therapies for children with DCM vary by prescriber, symptoms, and specific disease pathology. Therapeutics started at the time of initial pediatric DCM diagnosis showed anti-congestive therapy (82%), angiotensin-converting enzyme inhibitors (ACEi) (64%), anti-arrhythmics (38%), β-blockers (4%), and calcium channel blockers (3%), ECMO (3%), VAD (2%), pacemaker or balloon pump (1%) (3). A 2011 review of the PCMR revealed patients 1 month after diagnosis to be on anti-HF therapies, including digoxin, diuretics, or both, (84%), ACEi (66%), and β-blockers (4%) (12). Additionally, 16% were on IV inotropes 1 month following diagnosis (91). Fortunately, mortality rates of pediatric DCM have decreased over time as a result of new management paradigms (92). Recent research & drug development in adults with HFrEF has provided valuable insights into the pharmacological advantages of targeted therapies. This knowledge is derived from extensive, large-scale, randomized, placebo-controlled clinical trials, shedding light on the incidence of hospitalization, morbidity, and mortality associated with these treatments. These demonstrated ACEi/ARBs, angiotensin receptor/neprilysin inhibitors, β-blockers, mineralocorticoid receptor antagonists, ivabradine, sodium-glucose cotransporter 2 inhibitor to be significant reducers of hospitalization and/or mortality (93). Given the limitation in randomized-controlled trials in children, treatment strategies for children are largely extrapolated from information on effective treatments in adults. This extrapolation is built out of necessity for a standardized treatment plan and, in practice, has measured efficacy attributable to the differences in pediatric cardiac adaptation to DCM.

A few critical studies do exist examining pediatric DCM patient responses to therapeutic regimens. A randomized, double-blind, placebo-controlled trial of ivabradine therapy taken in combination with ACEi/ARBs (98%), mineralocorticoid receptor antagonists (79%), β-blockers (76%) among 116 children resulted in improved EF, reduced natriuretic peptide concentration, and reduction in heart rate (HR) compared to placebo (94). The PANORAMA-HF study demonstrated an initial greater reduction in natriuretic peptide levels with sacubitril-valsartan compared to ACEi, although this difference equilibrated at 12 months with no difference in outcome (95). No survival benefit was seen among digoxin & diuretics in the late 1970s or β-blockers, particularly carvedilol, in the early 2000s (96, 97). Meanwhile adults are known to improve with β-blockers therapy (98). This lack of strong data for the pharmacologic efficacy of β-blockers in pediatric DCM likely stems from the unique molecular adaptations of cardiomyocytes seen in children (97, 99, 100). Carvedilol, in combination with other medications, is known, however, to improve fractional shortening, reduce LV size, and decrease natriuretic peptide levels in children (101). This finding emphasizes the distinction between clinical outcomes & known physiologic changes stemming from surrogates of cardiac dysfunction. Another distinction between children and adult disease is seen with the use of phosphodiesterase-3 (PDE-3) inhibitors. Long-term treatment with PDE-3 inhibitors was noted to increase morbidity and mortality in adults (102, 103). In contrast, children with idiopathic DCM show a safe and effective response to these inhibitors. In fact, agents like milrinone are often employed acutely to bridge patients to oral therapies or transplantation (104, 105).

Physicians should always consider potential etiologies when designing patient-specific maintenance therapy protocols. When etiologies are known, pharmacotherapy can better target factors in molecular pathogenesis of DCM to achieve adequate treatment. Therapeutic approaches for acute myocarditis in children may include IVIG, corticosteroids, antivirals, or interferon- β, although data to suggest their effectiveness in preventing or treating pediatric DCM is limited (106–109). For pediatric DCM in patients DMD, a systemic review determined ACE inhibitors to be effective at limiting or prolonging onset of cardiac maladies in children (110). Antisense oligonucleotides programmed for genetic correction of underlying dystrophin mutation are also currently FDA-approved for DMD treatment (111). This strategy may influence further development of gene editing approaches to therapy for other etiologies. Dilated cardiomyopathy of mitochondrial origin may benefit from dietary supplements, including coenzyme Q10, creatine, L-carnitine, thiamine, riboflavin, folate, and other antioxidants such as vitamins C and E, however, the evidence to support these measures is not robust. In fact, a Cochrane meta-analysis concluded there is no clear evidence for their use (112, 113). Treatment of DCM related to primary carnitine deficiency, however, has been shown to respond to supplementing L-carnitine (114). Elamipretide is a medication that has shown some promise in improving stroke volume and functional ability for patients with dilated cardiomyopathy caused by Barth syndrome (115, 116). Analyses of the risks and benefits to therapy should be considered when administering cardiotoxic medications. Ameliorating the effects of doxorubicin, a cardiotoxic anthracycline often used acute lymphoblastic leukemia or Hodgkin disease, with dexrazoxane at the time of treatment has been shown to reduced cardiac-related complications, such as DCM, years later (117).

Transplantation is reserved as a definitive treatment for refractory DCM. The AHA outlines that children with stage D HF who are still symptomatic with maximal medical therapy qualify for continuous intravenous inotropic infusions, mechanical circulatory support, cardiac transplantation, or palliative/hospice care (118).

The treatment of pediatric DCM remains a complex challenge, marked by several limitations. A key limitation lies in the incomplete understanding of the biomolecular changes occurring in pediatric cardiomyocytes. Children are currently managed using protocols modeled on those for treating adults, with few therapies known to specifically benefit pediatric cases (9). This standard approach involves the use of diuretics, ACEi, β-adrenergic blockers, and aldosterone antagonists. In adults, the therapy goal is to minimize or reverse adverse cardiac remodeling, targeting features such as hypertrophy, myocardial fibrosis, inflammation, and capillary loss (119). However, evidence suggests that different pathogenic processes govern remodeling in children, involving varying levels of β-receptor, cAMP, connexin43, phosphatase, and phospholamban expression with distinct intracellular mRNA profiles and pathologic structural changes. The execution of clinical trials in children with heart conditions faces several barriers, including inadequate statistical power due to small sample sizes, phenotypic heterogeneity, limited observational periods, and age-specific variations in pharmacokinetics and pharmacodynamics. The ultimate objective is to uncover a more comprehensive genetic background for the disease, enabling the development of specific pathway-directed therapies. Promising studies, such as the REALM-DCM study for Lamin A/C gene mutation-associated DCM, and the use of oral p38 MAPK inhibitor ARRY-371797 in adults with symptomatic DCM, offer a glimpse into the potential for other targeted treatments in the future (120).

## 13 Monitoring disease

Routine monitoring of children with DCM is critical to track disease burden and prevent exacerbations. Echocardiography is the primary disease monitoring modality. The fractional shortening and EF are the most used metrics. These give insight into the contractile function of the heart, which would be impaired in DCM. Another metric used is LV posterior wall thickness. As the LV dilates, the LV posterior wall thins, contributing to poorer contractility (9). CMR is useful to monitor progression of disease given the detailed structural information it provides, although no standard techniques have been established. Biomarkers, such as NT-proBNP, have been proposed as surrogates for systolic function and cardiac remodeling in children. One study suggests they are useful in highly symptomatic children but have not been validated in pediatric patients with less severe DCM (121). The ISHLT (International Society for Heart and Lung Transplantation) also advises considering comprehensive or targeted genetic testing in specific situations. These tests can help identify mutations in genes, such as LMNA and SMN5, providing valuable insights into the genetic factors contributing to the condition and the potential risk within the affected individual's family (90).

## 14 Conclusion

Pediatric dilated cardiomyopathy represents a rare and primarily idiopathic condition associated with significant morbidity and mortality. The fundamental diagnostic approach requires matching clinical history & physical exam findings with echocardiographic parameters of LV dilation and dysfunction. The prognosis of disease varies based on age, indicators of left ventricular dysfunction, and etiological factors. While transplantation stands as a definitive therapy enhancing survival, its implementation entails inherent risks. Pharmacologic interventions, aligned with guidelines for adult heart failure with reduced ejection fraction, lack comprehensive validation through randomized controlled trials in the pediatric DCM population. Molecular pathways and histological cardiac responses further underscore the unique features of pediatric disease. Idiopathic cases, constituting approximately two-thirds of occurrences, are presumed to have genetic underpinnings. In certain cases, they can be considered pediatric manifestations of inherited cardiomyopathy syndromes caused by mutations in some of the same genes governing sarcomeric, nuclear, ion channel, transport, cytoskeletal, and extracellular matrix. However, recent studies indicate that infantile dilated cardiomyopathy may be caused by unique defects in the postnatal cardiac maturation, such as the failure of programmed centrosome breakdown in cardiomyocytes. A deeper comprehension of these intricacies holds the potential to inform targeted therapeutic approaches, thereby advancing outcomes in pediatric dilated cardiomyopathy.

## References

[1] Ashworth MT. Aspects of paediatric cardiovascular pathology. Diagn Histopathol. (2019) 25:313–23. 10.1016/j.mpdhp.2019.05.003

[2] Lipshultz SE Law YM Asante-Korang A Austin ED Dipchand AI Everitt MD Cardiomyopathy in children: classification and diagnosis: a scientific statement from the American heart association. Circulation. (2019) 140:9–68. 10.1161/CIR.0000000000000682 31132865

[3] Towbin JA Lowe AM Colan SD Sleeper LA Orav EJ Clunie S Incidence, causes, and outcomes of dilated cardiomyopathy in children. JAMA. (2006) 296:1867. 10.1001/jama.296.15.1867 17047217

[4] Lipshultz SE Sleeper LA Towbin JA Lowe AM Orav EJ Cox GF The incidence of pediatric cardiomyopathy in two regions of the United States. N Engl J Med. (2003) 348:1647–55. 10.1056/NEJMoa021715 12711739

[5] DiGiorgi PL Reel MS Thornton B Burton E Naka Y Oz MC. Heart transplant and left ventricular assist device costs. J Heart Lung Transplant. (2005) 24:200–4. 10.1016/j.healun.2003.11.397 15701438

[6] O’Connell JB Bristow MR. Economic impact of heart failure in the United States: time for a different approach. J Heart Lung Transplant. (1994) 13:S107–12. 7947865

[7] Tsatsopoulou A Protonotarios I Xylouri Z Papagiannis I Anastasakis A Germanakis I Cardiomyopathies in children: an overview. Hellenic J Cardiol. (2023) 72:43–56. 10.1016/j.hjc.2023.02.007 36870438

[8] Cox GF Sleeper LA Lowe AM Towbin JA Colan SD Orav EJ Factors associated with establishing a causal diagnosis for children with cardiomyopathy. Pediatrics. (2006) 118:1519–31. 10.1542/peds.2006-0163 17015543

[9] Lipshultz SE Cochran TR Briston DA Brown SR Sambatakos PJ Miller TL Pediatric cardiomyopathies: causes, epidemiology, clinical course, preventive strategies and therapies. Future Cardiol. (2013) 9:817–48. 10.2217/fca.13.66 24180540 PMC3903430

[10] Mestroni L Brun F Spezzacatene A Sinagra G Taylor MRG. Genetic causes of dilated cardiomyopathy. Prog Pediatr Cardiol. (2014) 37:13–8. 10.1016/j.ppedcard.2014.10.003 25584016 PMC4288017

[11] Nugent AW Daubeney PEF Chondros P Carlin JB Cheung M Wilkinson LC The epidemiology of childhood cardiomyopathy in Australia. N Engl J Med. (2003) 348:1639–46. 10.1056/NEJMoa021737 12711738

[12] Wilkinson JD Landy DC Colan SD Towbin JA Sleeper LA Orav EJ The pediatric cardiomyopathy registry and heart failure: key results from the first 15 years. Heart Fail Clin. (2010) 6:401–13. 10.1016/j.hfc.2010.05.002 20869642 PMC2946942

[13] Daubeney PEF Nugent AW Chondros P Carlin JB Colan SD Cheung M Clinical features and outcomes of childhood dilated cardiomyopathy. Circulation. (2006) 114:2671–8. 10.1161/CIRCULATIONAHA.106.635128 17116768

[14] Richardson P McKenna W Bristow M Maisch B Mautner B O’Connell J Report of the 1995 world health organization/international society and federation of cardiology task force on the definition and classification of cardiomyopathies. Circulation. (1996) 93:841–2. 10.1161/01.cir.93.5.841 8598070

[15] McMurray JJ V Adamopoulos S Anker SD Auricchio A Böhm M Dickstein K ESC guidelines for the diagnosis and treatment of acute and chronic heart failure 2012: the task force for the diagnosis and treatment of acute and chronic heart failure 2012 of the European Society of Cardiology. Developed in collaboration with the heart failure association (HFA) of the ESC. Eur J Heart Fail. (2012) 14:803–69. 10.1093/eurjhf/hfs105 22828712

[16] McNally EM Mestroni L. Dilated cardiomyopathy. Circ Res. (2017) 121:731–48. 10.1161/CIRCRESAHA.116.309396 28912180 PMC5626020

[17] Yilmaz A Gdynia H-J Baccouche H Mahrholdt H Meinhardt G Basso C Cardiac involvement in patients with becker muscular dystrophy: new diagnostic and pathophysiological insights by a CMR approach. J Cardiovasc Magn Reson. (2008) 10:50. 10.1186/1532-429X-10-50 18983659 PMC2585564

[18] Silva MC Meira ZMA Gurgel Giannetti J da Silva MM Campos AFO Barbosa M de M Myocardial delayed enhancement by magnetic resonance imaging in patients with muscular dystrophy. J Am Coll Cardiol. (2007) 49:1874–9. 10.1016/j.jacc.2006.10.078 17481447

[19] Hor KN Taylor MD Al-Khalidi HR Cripe LH Raman S V Jefferies JL Prevalence and distribution of late gadolinium enhancement in a large population of patients with duchenne muscular dystrophy: effect of age and left ventricular systolic function. J Cardiovasc Magn Reson. (2013) 15:107. 10.1186/1532-429X-15-107 24359596 PMC3896985

[20] Moscatelli S Leo I Bianco F Borrelli N Beltrami M Garofalo M The role of multimodality imaging in pediatric cardiomyopathies. J Clin Med. (2023) 12:4866. 10.3390/jcm12144866 37510983 PMC10381492

[21] Merlo M Gagno G Baritussio A Bauce B Biagini E Canepa M Clinical application of CMR in cardiomyopathies: evolving concepts and techniques. Heart Fail Rev. (2022) 28:77–95. 10.1007/s10741-022-10235-9 35536402 PMC9902331

[22] Hershberger RE Givertz MM Ho CY Judge DP Kantor PF McBride KL Genetic evaluation of cardiomyopathy—a heart failure society of America practice guideline. J Card Fail. (2018) 24:281–302. 10.1016/j.cardfail.2018.03.004 29567486 PMC9903357

[23] Michels V V Moll PP Miller FA Tajik AJ Chu JS Driscoll DJ The frequency of familial dilated cardiomyopathy in a series of patients with idiopathic dilated cardiomyopathy. N Engl J Med. (1992) 326:77–82. 10.1056/NEJM199201093260201 1727235

[24] Ackerman MJ Priori SG Willems S Berul C Brugada R Calkins H HRS/EHRA expert consensus statement on the state of genetic testing for the channelopathies and cardiomyopathies this document was developed as a partnership between the heart rhythm society (HRS) and the European heart rhythm association (EHRA). Heart Rhythm. (2011) 8:1308–39. 10.1016/j.hrthm.2011.05.020 21787999

[25] Post GR Brown JH. G protein-coupled receptors and signaling pathways regulating growth responses ^1^. FASEB J. (1996) 10:741–9. 10.1096/fasebj.10.7.8635691 8635691

[26] Daaka Y Luttrell LM Lefkowitz RJ. Switching of the coupling of the β2-adrenergic receptor to different G proteins by protein kinase A. Nature. (1997) 390:88–91. 10.1038/36362 9363896

[27] Miyamoto SD Stauffer BL Nakano S Sobus R Nunley K Nelson P Beta-adrenergic adaptation in paediatric idiopathic dilated cardiomyopathy. Eur Heart J. (2014) 35:33–41. 10.1093/eurheartj/ehs229 22843448 PMC3877432

[28] Chadjichristos CE Kavvadas P Dussaule JC Abed A Chatziantoniou C. Reversibility of renal fibrosis. In: Orlando G, Williams D, editors. Kidney Transplantation, Bioengineering, and Regeneration: Kidney Transplantation in the Regenerative Medicine Era. London, UK: Academic Press (2017). p. 1013–23. 10.1016/B978-0-12-801734-0.00073-4

[29] Klapproth E Kämmerer S El-Armouche A. Function and regulation of phosphatase 1 in healthy and diseased heart. Cell Signal. (2022) 90:110203. 10.1016/j.cellsig.2021.110203 34822978

[30] Frank K Kranias EG. Phospholamban and cardiac contractility. Ann Med. (2000) 32:572–8. 10.3109/07853890008998837 11127935

[31] Tatman PD Woulfe KC Karimpour-Fard A Jeffrey DA Jaggers J Cleveland JC Pediatric dilated cardiomyopathy hearts display a unique gene expression profile. JCI Insight. (2017) 2:e94249. 10.1172/jci.insight.94249 28724804 PMC5518568

[32] Woulfe KC Siomos AK Nguyen H SooHoo M Galambos C Stauffer BL Fibrosis and fibrotic gene expression in pediatric and adult patients with idiopathic dilated cardiomyopathy. J Card Fail. (2017) 23:314–24. 10.1016/j.cardfail.2016.11.006 27890770 PMC5380509

[33] Patel MD Mohan J Schneider C Bajpai G Purevjav E Canter CE Pediatric and adult dilated cardiomyopathy represent distinct pathological entities. JCI Insight. (2017) 2:e94382. 10.1172/jci.insight.94382 PMC5518561 28724792

[34] Nishikawa T Uto K Kanai S Oda H Kawamura S Nakanishi T Histopathological aspects of cardiac biopsy in pediatric patients with dilated cardiomyopathy. Pediatr Int. (2011) 53:350–3. 10.1111/j.1442-200X.2010.03250.x 20854285

[35] Singh TP Sleeper LA Lipshultz S Cinar A Canter C Webber SA Association of left ventricular dilation at listing for heart transplant with postlisting and early posttransplant mortality in children with dilated cardiomyopathy. Circ Heart Fail. (2009) 2:591–8. 10.1161/CIRCHEARTFAILURE.108.839001 19919984

[36] Mallavarapu A Taksande A. Dilated cardiomyopathy in children: early detection and treatment. Cureus. (2022) 14:e31111. 10.7759/cureus.31111 36475220 PMC9720092

[37] Hitawala G Jain E Castellanos L Garimella R Akku R Chamavaliyathil AK Pediatric chemotherapy drugs associated with cardiotoxicity. Cureus. (2021) 13:e19658. 10.7759/cureus.19658 34976454 PMC8679581

[38] Lee TM Hsu DT Kantor P Towbin JA Ware SM Colan SD Pediatric cardiomyopathies. Circ Res. (2017) 121:855–73. 10.1161/CIRCRESAHA.116.309386 28912187 PMC5657298

[39] Rampersaud E Siegfried JD Norton N Li D Martin E Hershberger RE. Rare variant mutations identified in pediatric patients with dilated cardiomyopathy. Prog Pediatr Cardiol. (2011) 31:39–47. 10.1016/j.ppedcard.2010.11.008 21483645 PMC3072577

[40] Carniel E Taylor MRG Sinagra G Di Lenarda A Ku L Fain PR α-myosin heavy chain. Circulation. (2005) 112:54–9. 10.1161/CIRCULATIONAHA.104.507699 15998695

[41] Daehmlow S Erdmann J Knueppel T Gille C Froemmel C Hummel M Novel mutations in sarcomeric protein genes in dilated cardiomyopathy. Biochem Biophys Res Commun. (2002) 298:116–20. 10.1016/S0006-291X(02)02374-4 12379228

[42] Rangrez AY Kilian L Stiebeling K Dittmann S Yadav P Schulze-Bahr E Data on the role of cardiac α-actin (ACTC1) gene mutations on SRF-signaling. Data Brief. (2020) 28:105071. 10.1016/j.dib.2019.105071 31921954 PMC6950782

[43] Chang AN Potter JD. Sarcomeric protein mutations in dilated cardiomyopathy. Heart Fail Rev. (2005) 10:225–35. 10.1007/s10741-005-5252-6 16416045

[44] Hershberger RE Pinto JR Parks SB Kushner JD Li D Ludwigsen S Clinical and functional characterization of TNNT2 mutations identified in patients with dilated cardiomyopathy. Circ Cardiovasc Genet. (2009) 2:306–13. 10.1161/CIRCGENETICS.108.846733 20031601 PMC2900844

[45] Landim-Vieira M Johnston JR Ji W Mis EK Tijerino J Spencer-Manzon M Familial dilated cardiomyopathy associated with a novel combination of compound heterozygous TNNC1 variants. Front Physiol. (2020) 10:1612. 10.3389/fphys.2019.01612 32038292 PMC6990120

[46] Kobayashi T Solaro RJ. Increased Ca2+ affinity of cardiac thin filaments reconstituted with cardiomyopathy-related mutant cardiac troponin I. J Biol Chem. (2006) 281:13471–7. 10.1074/jbc.M509561200 16531415

[47] Sjöblom B Salmazo A Djinović-Carugo K. α-actinin structure and regulation. Cell Mol Life Sci. (2008) 65:2688–701. 10.1007/s00018-008-8080-8 18488141 PMC11131806

[48] Verdonschot JAJ Vanhoutte EK Claes GRF Helderman-van den Enden ATJM Hoeijmakers JGJ Hellebrekers DMEI A mutation update for the FLNC gene in myopathies and cardiomyopathies. Hum Mutat. (2020) 41:1091–111. 10.1002/humu.24004 32112656 PMC7318287

[49] Spinozzi S Liu C Chen Z Feng W Zhang L Ouyang K Nexilin is necessary for maintaining the transverse-axial tubular system in adult cardiomyocytes. Circ Heart Fail. (2020) 13:e006935. 10.1161/CIRCHEARTFAILURE.120.006935 32635769 PMC7583668

[50] Bruyndonckx L Vogelzang JL Bugiani M Straver B Kuipers IM Onland W Childhood onset nexilin dilated cardiomyopathy: a heterozygous and a homozygous case. Am J Med Genet A. (2021) 185:2464–70. 10.1002/ajmg.a.62231 33949776 PMC8359989

[51] Lakdawala NK Funke BH Baxter S Cirino AL Roberts AE Judge DP Genetic testing for dilated cardiomyopathy in clinical practice. J Card Fail. (2012) 18:296–303. 10.1016/j.cardfail.2012.01.013 22464770 PMC3666099

[52] Kamisago M Sharma SD DePalma SR Solomon S Sharma P McDonough B Mutations in sarcomere protein genes as a cause of dilated cardiomyopathy. N Engl J Med. (2000) 343:1688–96. 10.1056/NEJM200012073432304 11106718

[53] Herman DS Lam L Taylor MRG Wang L Teekakirikul P Christodoulou D Truncations of titin causing dilated cardiomyopathy. N Engl J Med. (2012) 366:619–28. 10.1056/NEJMoa1110186 22335739 PMC3660031

[54] Taylor MRG Slavov D Gajewski A Vlcek S Ku L Fain PR Thymopoietin (lamina-associated polypeptide 2) gene mutation associated with dilated cardiomyopathy. Hum Mutat. (2005) 26:566–74. 10.1002/humu.20250 16247757

[55] Taylor MRG Fain PR Sinagra G Robinson ML Robertson AD Carniel E Natural history of dilated cardiomyopathy due to lamin A/C gene mutations. J Am Coll Cardiol. (2003) 41:771–80. 10.1016/S0735-1097(02)02954-6 12628721

[56] Hutchison CJ Alvarez-Reyes M Vaughan OA. Lamins in disease: why do ubiquitously expressed nuclear envelope proteins give rise to tissue-specific disease phenotypes? J Cell Sci. (2001) 114:9–19. 10.1242/jcs.114.1.9 11112685

[57] Wilson KL. The nuclear envelope, muscular dystrophy and gene expression. Trends Cell Biol. (2000) 10:125–9. 10.1016/S0962-8924(99)01708-0 10740265

[58] McNair WP Ku L Taylor MRG Fain PR Dao D Wolfel E SCN5A mutation associated with dilated cardiomyopathy, conduction disorder, and arrhythmia. Circulation. (2004) 110:2163–7. 10.1161/01.CIR.0000144458.58660.BB 15466643

[59] Schmitt JP Kamisago M Asahi M Li GH Ahmad F Mende U Dilated cardiomyopathy and heart failure caused by a mutation in phospholamban. Science (1979). (2003) 299:1410–3. 10.1126/science.1081578 12610310

[60] McNair WP Sinagra G Taylor MRG Di Lenarda A Ferguson DA Salcedo EE SCN5A mutations associate with arrhythmic dilated cardiomyopathy and commonly localize to the voltage-sensing mechanism. J Am Coll Cardiol. (2011) 57:2160–8. 10.1016/j.jacc.2010.09.084 21596231 PMC9689753

[61] Taylor MRG Slavov D Ku L Di Lenarda A Sinagra G Carniel E Prevalence of desmin mutations in dilated cardiomyopathy. Circulation. (2007) 115:1244–51. 10.1161/CIRCULATIONAHA.106.646778 17325244

[62] Vatta M Mohapatra B Jimenez S Sanchez X Faulkner G Perles Z Mutations in cypher/ZASPin patients with dilated cardiomyopathy and left ventricular non-compaction. J Am Coll Cardiol. (2003) 42:2014–27. 10.1016/j.jacc.2003.10.021 14662268

[63] Towbin JA Hejtmancik JF Brink P Gelb B Zhu XM Chamberlain JS X-linked dilated cardiomyopathy. Molecular genetic evidence of linkage to the duchenne muscular dystrophy (dystrophin) gene at the Xp21 locus. Circulation. (1993) 87:1854–65. 10.1161/01.CIR.87.6.1854 8504498

[64] Olson TM Illenberger S Kishimoto NY Huttelmaier S Keating MT Jockusch BM. Metavinculin mutations Alter actin interaction in dilated cardiomyopathy. Circulation. (2002) 105:431–7. 10.1161/hc0402.102930 11815424

[65] Murakami T Hayashi YK Noguchi S Ogawa M Nonaka I Tanabe Y Fukutin gene mutations cause dilated cardiomyopathy with minimal muscle weakness. Ann Neurol. (2006) 60:597–602. 10.1002/ana.20973 17036286

[66] Knöll R Postel R Wang J Krätzner R Hennecke G Vacaru AM Laminin-α4 and integrin-linked kinase mutations cause human cardiomyopathy via simultaneous defects in cardiomyocytes and endothelial cells. Circulation. (2007) 116:515–25. 10.1161/CIRCULATIONAHA.107.689984 17646580

[67] Norgett EE. Recessive mutation in desmoplakin disrupts desmoplakin-intermediate filament interactions and causes dilated cardiomyopathy, woolly hair and keratoderma. Hum Mol Genet. (2000) 9:2761–6. 10.1093/hmg/9.18.2761 11063735

[68] Chun YW Miyamoto M Williams CH Neitzel LR Silver-Isenstadt M Cadar AG Impaired reorganization of centrosome structure underlies human infantile dilated cardiomyopathy. Circulation. (2023) 147:1291–303. 10.1161/CIRCULATIONAHA.122.060985 36970983 PMC10133173

[69] Arimura T Ishikawa T Nunoda S Kawai S Kimura A. Dilated cardiomyopathy-associated BAG3 mutations impair Z-disc assembly and enhance sensitivity to apoptosis in cardiomyocytes. Hum Mutat. (2011) 32:1481–91. 10.1002/humu.21603 21898660

[70] Feldman AM Begay RL Knezevic T Myers VD Slavov DB Zhu W Decreased levels of BAG3 in a family with a rare variant and in idiopathic dilated cardiomyopathy. J Cell Physiol. (2014) 229:1697–702. 10.1002/jcp.24615 24623017 PMC4296028

[71] Koelemen J Gotthardt M Steinmetz LM Meder B. RBM20-related cardiomyopathy: current understanding and future options. J Clin Med. (2021) 10:4101. 10.3390/jcm10184101 34575212 PMC8468976

[72] Bowles NE Bowles KR Towbin JA. The “final common pathway” hypothesis and inherited cardiovascular disease. Herz. (2000) 25:168–75. 10.1007/s000590050003 10904835

[73] Mehdiabadi NR Choon B Sim B Phipson B Kalathur RKR Sun Y Defining the fetal gene program at single-cell resolution in pediatric dilated cardiomyopathy. Circulation. (2022) 146:1105–8. 10.1161/CIRCULATIONAHA.121.057763 36191067 PMC9528943

[74] Khan RS Pahl E Dellefave-Castillo L Rychlik K Ing A Yap KL Genotype and cardiac outcomes in pediatric dilated cardiomyopathy. J Am Heart Assoc. (2022) 11:e022854. 10.1161/JAHA.121.022854/FORMAT/EPUB PMC9075202 34935411

[75] van der Meulen MH Herkert JC den Boer SL du Marchie Sarvaas GJ Blom N ten Harkel ADJ Genetic evaluation of A nation-wide Dutch pediatric DCM cohort: the use of genetic testing in risk stratification. Circ Genom Precis Med. (2022) 15:e002981. 10.1161/CIRCGEN.120.002981 36178741 PMC9622377

[76] Kannan S Kwon C. Regulation of cardiomyocyte maturation during critical perinatal window. J Physiol. (2020) 598:2941–56. 10.1113/JP276754 30571853 PMC7682257

[77] Uosaki H Cahan P Lee DI Wang S Miyamoto M Fernandez L Transcriptional landscape of cardiomyocyte maturation. Cell Rep. (2015) 13:1705–16. 10.1016/j.celrep.2015.10.032 26586429 PMC4662925

[78] Kannan S Farid M Lin BL Miyamoto M Kwon C. Transcriptomic entropy benchmarks stem cell-derived cardiomyocyte maturation against endogenous tissue at single cell level. PLoS Comput Biol. (2021) 17:e1009305. 10.1371/JOURNAL.PCBI.1009305 34534204 PMC8448341

[79] Guo Y Jardin BD Zhou P Sethi I Akerberg BN Toepfer CN Hierarchical and stage-specific regulation of murine cardiomyocyte maturation by serum response factor. Nat Commun. (2018) 9:1–16. 10.1038/s41467-018-06347-2 30242271 PMC6155060

[80] Shenje LT Andersen P Halushka MK Lui C Fernandez L Collin GB Mutations in alström protein impair terminal differentiation of cardiomyocytes. Nat Commun. (2014) 5:1–11. 10.1038/ncomms4416 PMC3992616 24595103

[81] Everitt MD Sleeper LA Lu M Canter CE Pahl E Wilkinson JD Recovery of echocardiographic function in children with idiopathic dilated cardiomyopathy. J Am Coll Cardiol. (2014) 63:1405–13. 10.1016/j.jacc.2013.11.059 24561146 PMC4030008

[82] Alvarez JA Wilkinson JD Lipshultz SE. Outcome predictors for pediatric dilated cardiomyopathy: a systematic review. Prog Pediatr Cardiol. (2007) 23:25–32. 10.1016/j.ppedcard.2007.05.009 19701490 PMC2729918

[83] Azevedo VMP Santos MA Albanesi Filho FM Castier MB Tura BR Amino JGC. Outcome factors of idiopathic dilated cardiomyopathy in children—a long-term follow-up review. Cardiol Young. (2007) 17:175–84. 10.1017/S1047951107000170 17244382

[84] Pietra BA Kantor PF Bartlett HL Chin C Canter CE Larsen RL Early predictors of survival to and after heart transplantation in children with dilated cardiomyopathy. Circulation. (2012) 126:1079–86. 10.1161/CIRCULATIONAHA.110.011999 22800850 PMC3510785

[85] Pahl E Sleeper LA Canter CE Hsu DT Lu M Webber SA Incidence of and risk factors for sudden cardiac death in children with dilated cardiomyopathy. J Am Coll Cardiol. (2012) 59:607–15. 10.1016/j.jacc.2011.10.878 22300696 PMC3280885

[86] Larsen RL Canter CE Naftel DC Tressler M Rosenthal DN Blume ED The impact of heart failure severity at time of listing for cardiac transplantation on survival in pediatric cardiomyopathy. J Heart Lung Transplant. (2011) 30:755–60. 10.1016/j.healun.2011.01.718 21419658 PMC3110638

[87] Tsirka AE Trinkaus K Chen S-C Lipshultz SE Towbin JA Colan SD Improved outcomes of pediatric dilated cardiomyopathy with utilization of heart transplantation. J Am Coll Cardiol. (2004) 44:391–7. 10.1016/j.jacc.2004.04.035 15261937

[88] Rath A Weintraub R. Overview of cardiomyopathies in childhood. Front Pediatr. (2021) 9:708732. 10.3389/fped.2021.708732 34368032 PMC8342800

[89] Singh RK Canter CE Shi L Colan SD Dodd DA Everitt MD Survival without cardiac transplantation among children with dilated cardiomyopathy. J Am Coll Cardiol. (2017) 70:2663–73. 10.1016/j.jacc.2017.09.1089 29169474 PMC5726578

[90] Kirk R Dipchand AI Rosenthal DN Addonizio L Burch M Chrisant M The international society for heart and lung transplantation guidelines for the management of pediatric heart failure: executive summary. J Heart Lung Transplant. (2014) 33:888–909. 10.1016/j.healun.2014.06.002 25110323

[91] Harmon WG Sleeper LA Cuniberti L Messere J Colan SD Orav EJ Treating children with idiopathic dilated cardiomyopathy (from the pediatric cardiomyopathy registry). Am J Cardiol. (2009) 104:281–6. 10.1016/j.amjcard.2009.03.033 19576361 PMC2746101

[92] Stidham J Feingold B Almond CS Burstein DS Krack P Price JF Establishing baseline metrics of heart failure medication use in children: a collaborative effort from the ACTION network. Pediatr Cardiol. (2021) 42:315–23. 10.1007/s00246-020-02485-x 33044586

[93] Bogle C Colan SD Miyamoto SD Choudhry S Baez-Hernandez N Brickler MM Treatment strategies for cardiomyopathy in children: a scientific statement from the American heart association. Circulation. (2023) 148:174–95. 10.1161/CIR.0000000000001151 37288568

[94] Bonnet D Berger F Jokinen E Kantor PF Daubeney PEF. Ivabradine in children with dilated cardiomyopathy and symptomatic chronic heart failure. J Am Coll Cardiol. (2017) 70:1262–72. 10.1016/j.jacc.2017.07.725 28859790

[95] Shaddy R Canter C Halnon N Kochilas L Rossano J Bonnet D Design for the sacubitril/valsartan (LCZ696) compared with enalapril study of pediatric patients with heart failure due to systemic left ventricle systolic dysfunction (PANORAMA-HF study). Am Heart J. (2017) 193:23–34. 10.1016/j.ahj.2017.07.006 29129252

[96] Kantor PF Abraham JR Dipchand AI Benson LN Redington AN. The impact of changing medical therapy on transplantation-free survival in pediatric dilated cardiomyopathy. J Am Coll Cardiol. (2010) 55:1377–84. 10.1016/j.jacc.2009.11.059 20338500

[97] Shaddy RE Boucek MM Hsu DT Boucek RJ Canter CE Mahony L Carvedilol for children and adolescents with heart failure. JAMA. (2007) 298:1171. 10.1001/jama.298.10.1171 17848651

[98] Konstam MA. Improving clinical outcomes with drug treatment in heart failure: what have trials taught? Am J Cardiol. (2003) 91:9–14. 10.1016/S0002-9149(02)03374-X 12670637

[99] Adorisio R Cantarutti N Ciabattini M Amodeo A Drago F. Real-world use of carvedilol in children with dilated cardiomyopathy: long-term effect on survival and ventricular function. Front Pediatr. (2022) 10:845406. 10.3389/fped.2022.845406 PMC9010785 35433536

[100] Vaidyanathan B. Is there a role for carvedilol in the management of pediatric heart failure? A meta analysis and e-mail survey of expert opinion. Ann Pediatr Cardiol. (2009) 2:74. 10.4103/0974-2069.52816 20300274 PMC2840768

[101] Petko C Minich LL Everitt MD Holubkov R Shaddy RE Tani LY. Echocardiographic evaluation of children with systemic ventricular dysfunction treated with carvedilol. Pediatr Cardiol. (2010) 31:780–4. 10.1007/s00246-010-9700-2 20390261

[102] Cuffe MS. Short-term intravenous milrinone for acute exacerbation of chronic heart failure A randomized controlled trial. JAMA. (2002) 287:1541. 10.1001/jama.287.12.1541 11911756

[103] Felker GM Benza RL Chandler AB Leimberger JD Cuffe MS Califf RM Heart failure etiology and response tomilrinone in decompensated heart failure. J Am Coll Cardiol. (2003) 41:997–1003. 10.1016/S0735-1097(02)02968-6 12651048

[104] Berg A M Snell L Mahle W T. Home inotropic therapy in children. J Heart Lung Transplant. (2007) 26:453–7. 10.1016/j.healun.2007.02.004 17449413

[105] Price JF Towbin JA Dreyer WJ Moffett BS Kertesz NJ Clunie SK Outpatient continuous parenteral inotropic therapy as bridge to transplantation in children with advanced heart failure. J Card Fail. (2006) 12:139–43. 10.1016/j.cardfail.2005.11.001 16520263

[106] Kühl U Pauschinger M Schwimmbeck PL Seeberg B Lober C Noutsias M Interferon-β treatment eliminates cardiotropic viruses and improves left ventricular function in patients with myocardial persistence of viral genomes and left ventricular dysfunction. Circulation. (2003) 107:2793–8. 10.1161/01.CIR.0000072766.67150.51 12771005

[107] Schultheiss H-P Piper C Sowade O Waagstein F Kapp J-F Wegscheider K Betaferon in chronic viral cardiomyopathy (BICC) trial: effects of interferon-β treatment in patients with chronic viral cardiomyopathy. Clin Res Cardiol. (2016) 105:763–73. 10.1007/s00392-016-0986-9 27112783

[108] Law YM Lal AK Chen S Čiháková D Cooper LT Deshpande S Diagnosis and management of myocarditis in children. Circulation. (2021) 144:e123–35. 10.1161/CIR.0000000000001001 34229446

[109] Canter CE Simpson KE. Diagnosis and treatment of myocarditis in children in the current era. Circulation. (2014) 129:115–28. 10.1161/CIRCULATIONAHA.113.001372 24396015

[110] Bourke JP Bueser T Quinlivan R. Interventions for preventing and treating cardiac complications in duchenne and becker muscular dystrophy and X-linked dilated cardiomyopathy. Cochrane Database Syst Rev. (2018) 10:CD009068. 10.1002/14651858.CD009068.pub3 30326162 PMC6517009

[111] Johnston JR McNally EM. Genetic correction strategies for duchenne muscular dystrophy and their impact on the heart. Prog Pediatr Cardiol. (2021) 63:101460. 10.1016/j.ppedcard.2021.101460 34898968 PMC8656413

[112] Meyers DE Basha HI Koenig MK. Mitochondrial cardiomyopathy: pathophysiology, diagnosis, and management. Tex Heart Inst J. (2013) 40:385–94. . 24082366 PMC3783139

[113] Pfeffer G Majamaa K Turnbull DM Thorburn D Chinnery PF. Treatment for mitochondrial disorders. Cochrane Database Syst Rev. (2012) 2012:CD004426. 10.1002/14651858.CD004426.pub3 22513923 PMC7201312

[114] Fu L Huang M Chen S. Primary carnitine deficiency and cardiomyopathy. Korean Circ J. (2013) 43:785. 10.4070/kcj.2013.43.12.785 24385988 PMC3875693

[115] Thompson W R Hornby B Manuel R Bradley E Laux J Carr J A phase 2/3 randomized clinical trial followed by an open-label extension to evaluate the effectiveness of elamipretide in barth syndrome, a genetic disorder of mitochondrial cardiolipin metabolism. Genet Med. (2021) 23:471–8. 10.1038/s41436-020-01006-8 33077895 PMC7935714

[116] Sabbah HN. Elamipretide for barth syndrome cardiomyopathy: gradual rebuilding of a failed power grid. Heart Fail Rev. (2022) 27:1911–23. 10.1007/s10741-021-10177-8 34623544 PMC9388406

[117] Chow EJ Aggarwal S Doody DR Aplenc R Armenian SH Baker KS Dexrazoxane and long-term heart function in survivors of childhood cancer. J Clin Oncol. (2023) 41:2248–57. 10.1200/JCO.22.02423 36669148 PMC10448941

[118] Yancy CW Jessup M Bozkurt B Butler J Casey DE Drazner MH 2013 ACCF/AHA guideline for the management of heart failure. Circulation. (2013) 128:e147–e239. 10.1161/CIR.0b013e31829e8776 23747642

[119] Burchfield JS Xie M Hill JA. Pathological ventricular remodeling. Circulation. (2013) 128:388–400. 10.1161/CIRCULATIONAHA.113.001878 23877061 PMC3801217

[120] Judge DP Lakdawala NK Taylor MRG Mestroni L Li H Oliver C Long-term efficacy and safety of ARRY-371797 (PF-07265803) in patients with lamin A/C–related dilated cardiomyopathy. Am J Cardiol. (2022) 183:93–8. 10.1016/j.amjcard.2022.08.001 36114020

[121] Rusconi PG Ludwig DA Ratnasamy C Mas R Harmon WG Colan SD Serial measurements of serum NT-proBNP as markers of left ventricular systolic function and remodeling in children with heart failure. Am Heart J. (2010) 160:776–83. 10.1016/j.ahj.2010.07.012 20934575 PMC2964279

