# Supplementary material for: Pediatric dilated cardiomyopathy: a review of current clinical approaches and pathogenesis
Source: Front Pediatr. 2024 Jun 19;12:1404942. doi: 10.3389/fped.2024.1404942 (PMC11223501; doi:10.3389/fped.2024.1404942)
Supplement: Supplementary file 1 [file Table1.docx]

Supplementary Material

# Supplementary Table S1A-C: (A) Pharmacologic Therapy, (B) Electrophysiologic Interventions, (C) Mechanical Circulatory Support for Pediatric HFrEF – ISHLT, AEPC, PACES guidelines

| Modality/Therapy | | Guidelines (by NYHA class) | Level of Evidence |
| --- | --- | --- | --- |
| Pharmacologic | | | |
|  | Diuretics | Class I:  Fluid retention & ventricular dysfunction | C |
|  | Angiotensin converting enzyme inhibitors (ACEi) | Class I:  Symptomatic LV dysfunction  Class IIa:  Asymptomatic LV dysfunction  Diagnosis of DMD  Class IIb:  Not used for single ventricle congenital heart disease except in specific circumstances (valvular regurgitation, ventricular dysfunction) | B  B  B  B |
|  | Angiotensin II Receptor Blockers (ARB) | Class IIa:  Systemic ventricular systolic dysfunction but are intolerant of ACE inhibitors | C |
|  | β-blockers | Class IIa:  Symptomatic with systemic LV systolic dysfunction  Asymptomatic with systemic LV systolic dysfunction | B  B |
|  | Mineralocorticoid Antagonists | Class I:  Systemic LV dysfunction | C |
|  | Digoxin and cardiac glycosides | Class I:  Not recommended if asymptomatic  Class IIa:  Symptomatic relief* | C  C |
|  | Hydralazine combination with isosorbide dinitrate | Class III:  Not recommended | C |
|  | Anti-arrhythmic medications | Class IIb:  Persistent poorly tolerated arrhythmias after normalization of electrolyte disturbances & metabolic issues  Class III:  Not recommended | C  C |
|  | Statin | Class III:  Not recommended | C |
|  | Renin inhibitor | Class III:  Not recommended | C |
|  | Anticoagulation (heparin, warfarin) | Class I:  Presence of intracardiac thrombus  Class IIa:  History of thrombus or thromboembolic event with EF < 25% or fractional shortening < 15%  Class III:  Not recommended unless history of thrombus or thromboembolic event | B  C  C |
|  | Nesiritide | Class IIb:  Not recommended unless central venous pressure unable to be lowered by other therapies | C |
|  | Inotropic agents | Class IIa:  Symptomatic relief in palliative setting  Class III:  Not recommended unless a bridge to transplant | C  C |
|  | Vasopressin | Class II:  Not recommended | C |
| *Goal concentration of 0.5-0.9 ng/mL with close attention if on amiodarone, carvedilol, or risk of renal dysfunction | | | |
| Table 2A: Pharmacologic Therapy for pediatric HFrEF – ISHLT, AEPC, PACES guidelines | | | |
| Modality/Therapy | | Guidelines (by NYHA class) | Level of Evidence |
| Electrophysiology Interventions | | | |
|  | Pacemaker | Class I:  2^nd^ or 3^rd^ degree atrioventricular block with ventricular dysfunction  Class IIa:  LV apical pacing useful in epicardial pacing systems | B  B |
|  | Cardiac resynchronization therapies | Class IIa:  Systemic EF < 35%, complete bundle branch block pattern, QRS duration (native or paced) > ULN for age, NYHA class II-IV on GDMT  Class IIb:  Systemic RV with EF < 35%, complete right bundle branch block pattern, QRS duration (native or paced) > ULN for age, NYHA Class II-IV on GDMT  Single ventricle with EF < 35%, complete right bundle branch block pattern, QRS duration (native or paced) > ULN for age, NYHA Class II-IV on GDMT | B  C  C |
|  | ICD Therapy | Class I:  Survived cardiac arrest with work-up to exclude reversible causes  Class IIa:  Unexplained syncope with moderate LV dysfunction  Class IIb:  EF <35% & NYHA II or III  Syncope in presence of ventricular dysfunctions  Non-sustained or sustained ventricular tachycardia requiring a VAD | B  C  C  C  C |
|  | Ablation Therapy | Class I:  Tachycardia-induced cardiomyopathy when medical management fails  Class IIa:  Tachycardia-induced cardiomyopathy therapy  Class IIb:  PVCs & idiopathic cardiomyopathy when medical management fails | B  B  B |
| Table 2B: Electrophysiology Interventions for pediatric HFrEF – ISHLT, AEPC, PACES guidelines | | | |
|  | | | |
| Modality/Therapy | | Guidelines (by NYHA class) | Level of Evidence |
| Mechanical Circulatory Support | | | |
|  | Durable VAD device | Class I:  Bridge to transplant with failing inotropic support & early, reversible dysfunction of one other organ system  Class IIa:  Management by appropriate specialist on VADs  Class IIb:  Destination therapy if not transplant-eligible | C  C  C |
|  | ECMO | Class IIa:  Bridge to recovery of function in cardiac arrest or cardiogenic shock with pulmonary compromise  Bridge to recovery of function in isolated cardiac failure believed to be reversible  Cardiogenic shock not believed to be reversible for resuscitation of end-organ dysfunction | C  C  C |
|  | BiVAD | Class IIb:  Failed LVAD | C |
| Table 2A, 2B, 2C abbreviations: NYHA: New York Heart association, DMD: Duchenne Muscular Dystrophy, ULN: upper limit of normal, GDMT: goal-directed medical therapy, ICD: Implantable cardioverter-defibrillators, (L)VAD: (Left) Ventricular assist device, ECMO: Extracorporeal membrane oxygenation, ISHLT: International Society for Heart and Lung Transplantation, AEPC: Association of European Pediatric and Congenital Cardiology, PACES: Pediatric and Congenital Electrophysiology Society | | | |
| Table 2C: Mechanical Circulatory Support for pediatric HFrEF – ISHLT, AEPC, PACES guidelines | | | |
|  | | | |
